# Autophagy as a Guardian of Vascular Niche Homeostasis

**DOI:** 10.3390/ijms251810097

**Published:** 2024-09-20

**Authors:** Konstantin Dergilev, Alexandre Gureenkov, Yelena Parfyonova

**Affiliations:** 1 National Medical Research Centre of Cardiology Named after Academician E.I. Chazov, 121552 Moscow, Russiaevparfenova@cardio.ru (Y.P.); 2 Faculty of Fundamental Medicine, Lomonosov Moscow State University, 119991 Moscow, Russia

**Keywords:** autophagy, vascular niche, vascular regeneration

## Abstract

The increasing burden of vascular dysfunction on healthcare systems worldwide results in higher morbidity and mortality rates across pathologies, including cardiovascular diseases. Vasculopathy is suggested to be caused by the dysregulation of vascular niches, a microenvironment of vascular structures comprising anatomical structures, extracellular matrix components, and various cell populations. These elements work together to ensure accurate control of the vascular network. In recent years, autophagy has been recognized as a crucial regulator of the vascular microenvironment responsible for maintaining basic cell functions such as proliferation, differentiation, replicative senescence, and apoptosis. Experimental studies indicate that autophagy activation can be enhanced or inhibited in various pathologies associated with vascular dysfunction, suggesting that autophagy plays both beneficial and detrimental roles. Here, we review and assess the principles of autophagy organization and regulation in non-tumor vascular niches. Our analysis focuses on significant figures in the vascular microenvironment, highlighting the role of autophagy and summarizing evidence that supports the systemic or multiorgan nature of the autophagy effects. Finally, we discuss the critical organizational and functional aspects of the vasculogenic niche, specifically in relation to autophagy. The resulting dysregulation of the vascular microenvironment contributes to the development of vascular dysfunction.

## 1. Introduction

The vascular system is a complex network of interconnected vessels providing blood circulation in the body, responsible for delivering oxygen and nutrients and eliminating exchange products [1]. The central element of these vessels is the endothelium, which acts as an interface between the blood and cells surrounding the vessel wall [2,3]. Various biological stimuli elicit a complex response in the vascular microenvironment, known as the vascular niche, enabling the dynamic regulation of vascular function. Ruget and Ebert were the first to identify the population of vascular cells differing from the endothelium during the late nineteenth century [4,5]. The remarkable diversity in this population is evidenced by a wide range of adult stem cells and mesenchymal-like cells identified in the walls of arteries, veins, and capillaries in various organs [6]. Over time, the understanding of a vascular niche has broadened to encompass the microenvironment surrounding a blood vessel, including anatomical structures, components of the extracellular matrix, and different cell populations. Within the niche, there are minor populations of stem cells, endothelial cells (ECs), and other cell types and stroma. These cells are responsible for releasing specific signals critical for maintaining specific cell functions [7]. This function is largely realized by vascular cells of three types: vascular smooth muscle cells (SMCs), pericytes, and fibroblasts. While SMCs surround large vessels such as arteries and veins and are separated from ECs by the basement membrane, pericytes surround smaller vessels and capillaries and are in direct contact with ECs [8,9,10]. The Kramman group employed single-cell analysis to identify distinct cell populations within the cardiac vascular niche [11]. These included fibroblasts (Dcn+, Col1a1+, and Pdgfrα+), ECs (Kdr+ and Pecam1+), and mural cells (Rgs5+, Kcnj8+, and Vtn+). The mural cells were subdivided into pericytes (Abcc9+ and Colec11+) and smooth muscle cells of the vascular wall (Acta2+ and Tagln+). Furthermore, the researchers identified several minor clusters, classified as Schwann cells (Kcna1+ and Plp1+), lymphatic ECs (Ccl21a+ and Mmrn1+), and proliferating ECs (Top2a+ and Pecam1+). The vascular niche is a special type of microenvironment that serves as a milieu for stem cells and possesses distinct anatomical and functional characteristics, ensuring the formation of specific signals to provide self-maintenance of the stem and surrounding cells, control of their number, formation, and fate of their progeny. One of the most studied vascular microenvironments is the bone marrow niche, in which hematopoietic stem cells are located. The vascular niche of bone marrow consists of a network of fine-walled sinusoidal vessels, the integrity of which is maintained by components of the extracellular matrix and surrounding vascular cells, which is the microenvironment for hematopoietic stem cells [12,13,14,15,16]. The findings of Professor Morrison and his colleagues demonstrated the intricate relationship between hematopoietic stem cells in the bone marrow and the endothelium of sinusoids, thus regulating their quiescent state [17,18,19,20,21]. These findings confirm the concept of a vascular niche. Later, niches with similar organization principles were found in other organs, including the brain [22], lungs [23], skin [24], and heart [11]. These data confirm that the vascular niche exists as a unique dynamic system, shaping the foundation for renewal and maintenance of tissue homeostasis, with the violation of its regulation leading to the development of pathology. An indicative example of this pathology is cardiac vasculopathy. Coronary microvascular dysfunction is a pathological condition that affects the structure and function of the coronary microcirculation, resulting in impaired coronary blood flow and eventual myocardial ischemia [25]. The underlying pathology encompasses several factors, including endothelial dysfunction, microvascular remodeling, phenotype conversion, lumen obstruction, and others. Therefore, searching for effective targets for correction of perivascular niche homeostasis may provide a basis for treating vascular dysfunction. A novel paradigm related to autophagy has recently emerged, indicating its involvement in various chronic diseases associated with microvascular dysfunction.

In this review, we first define autophagy and then explore its role in the non-tumor vascular niche regulation and development/progression of vascular dysfunction.

## 2. Autophagy Machinery

**Figure 1:**
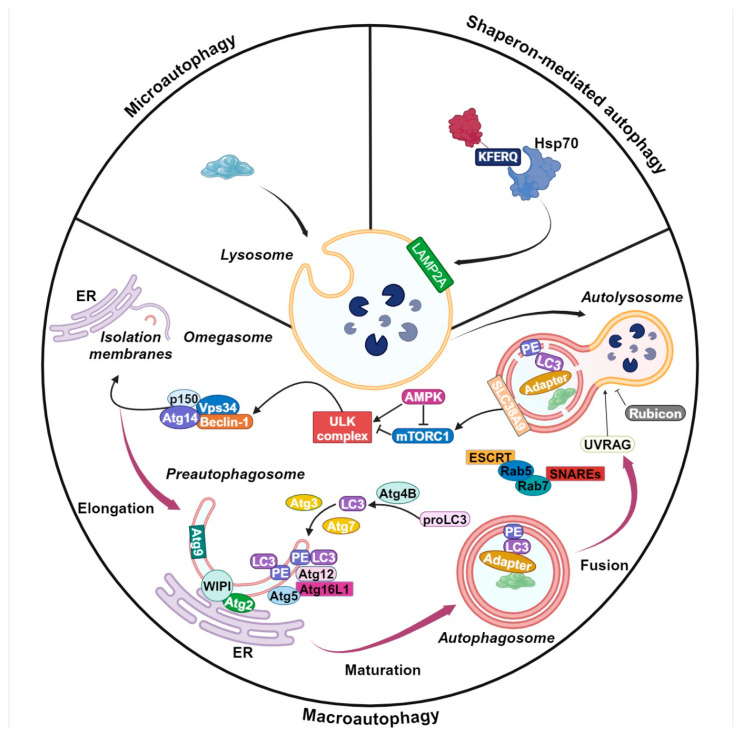
Basic mechanisms of autophagy. Microautophagy involves the direct engulfment of cytoplasmic components into lysosomes. Chaperone-mediated autophagy specifically targets and degrades proteins with a KFERQ motif. These proteins are recognized by the cytoplasmic chaperone Hsp70 and subsequently transported into the lysosome via LAMP2A. Macroautophagy, more commonly referred to as autophagy, involves the sequestration of cytoplasmic components within a double-membrane structure known as the autophagosome, which subsequently fuses with the lysosome for degradation. Autophagosome formation and maturation necessitate the involvement of several key protein complexes. The activation of the ULK complex is a critical initial step involving the phosphorylation of downstream ATG proteins. Autophagy is commonly initiated by nutrient deprivation mediated by AMPK and mTORC1. AMPK activates autophagy, while mTORC1 suppresses the ULK complex through phosphorylation. Following ULK activation, the PI3K complex, comprising Vps34, p150, Atg14, and Beclin-1, is essential for omegasome formation and PI3P generation. PI3P recruits WIPI, Atg9, and Atg2 proteins, forming a structure for elongating pre-autophagosome membranes. Maturation involves two ubiquitin-like conjugation systems: the Atg5/Atg12/Atg16L1 complex and the LC3 protein complex. LC3, processed from proLC3 to LC3 I by Atg4B and then lipidated to LC3 II, integrates into the autophagosome membrane. Finally, the mature autophagosome fuses with the lysosome to form the autolysosome. This fusion is regulated by SNAREs, ESCRT, and Rab GTPases, with Vps34/p150/Beclin-1 and UVRAG promoting fusion and Rubicon inhibiting autophagy. Created with BioRender.com (https://app.biorender.com accessed on 21 August 2024).

Autophagy is commonly classified into three forms: microautophagy, chaperon-mediated autophagy, and macroautophagy [26,27]. Microautophagy refers to the direct uptake of cytosolic components by the lysosome through invagination of its membrane [28]. During chaperone-mediated autophagy, proteins carrying a specific KFERQ sequence are recognized by the chaperone Hsp70 [29]. The recognition and binding of the chaperone complex with the lysosomal membrane protein Lamp2 occurs on the surface of lysosomes, allowing the translocation of the target protein across the lysosomal membrane. Macroautophagy (hereafter referred to as autophagy) is the most well-known and extensively researched type of autophagy [27,30]. It is characterized by the sequestration of a portion of the cytoplasm, including injured organelles or cellular debris, into an encapsulated double membrane vacuole known as an autophagosome. The process of autophagosome formation is driven by the concerted action of a suite of proteins referred to as ATG or ‘autophagy-related’ proteins [31]. Once formed, the autophagosome fuses with lysosomes to produce an autolysosome. In this environment, the contents of the autophagosome (proteins, lipids, nucleic acids, organelles, and others) are cleaved by hydrolases to produce nutrients to be exported to the cytosol for reuse [32]. Autophagy can be selective or non-selective regarding the recognition and degradation of cargo [33]. The selectivity is determined by the interaction of autophagy substrates and autophagy receptors, including chaperones and other general autophagy-related proteins. This process is aimed at eliminating specific substrates. For example, misshapen proteins are targeted for degradation via aggrephagy, while disrupted mitochondria are eliminated by mitophagy. Similarly, peroxisomes are degraded by pexophagy, the endoplasmic reticulum by reticulophagy, and ribosomes by ribophagy [34]. Although the precise molecular mechanism of selective autophagy remains elusive, the distinctive selective nature of this process has garnered significant interest as a potential focus for investigation in the context of various autophagy-related diseases and interventions. In contrast, the mechanisms of the non-selective variant of this process have been extensively studied. The macroautophagic process is divided into four essential stages: initiation, nucleation, maturation, and degradation [27]. Autophagosome formation is initiated by the generation of an isolation membrane, also referred to as a phagophore. This membrane is typically derived from endoplasmic reticulum (ER) membranes, although it may also originate from the Golgi, mitochondrial, or plasma membranes [35,36]. The formation of the isolation membrane is promoted by the Unc-51-like kinase 1 (ULK1)-autophagy-related gene 13 (ATG13)-family interacting protein 200 kD (FIP200) kinase complex, which is activated through coordinated regulation by the mechanistic target of rapamycin complex 1 (mTORC1) and AMP-activated protein kinase (AMPK) [37,38,39]. In response to elevated cytoplasmic AMP levels and/or reduced ATP production, AMPK is activated by specific upstream kinases that target threonine residues (Thr172), namely LKB1 and CaMKKβ. This activation results in the mTORC1 complex inhibition followed by autophagy initiation. AMPK inhibits mTORC1 by phosphorylating two proteins: TSC2, with serine residues different from those targeted by other upstream kinases, and Raptor, a partner of mTOR [40]. Recent studies have provided evidence of a direct correlation between the AMPK-activated autophagy and the ATG protein. Further research has demonstrated that, in the absence of glucose, AMPK activates autophagy by phosphorylating Ulk1 at the Ser317 and Ser777 sites, resulting in its activation. The Ulk complex, in turn, triggers the assembly of another macromolecular protein structure, comprising Beclin-1, VPS34 (a class III phosphatidylinositol 3-kinase (PI3K)), and additional proteins including Atg14L, Vps34, and Vps15 [41] during the nucleation phase of autophagy. The phosphorylation of Beclin-1 at Serine 14 by Ulk1 results in the activation of the Vps34 complex [42]. This complex is essential for the nascent autophagosome expansion through the formation of phosphatidylinositol 3-phosphate (PI3P). The presence of PI3P facilitates the translocation of multiple autophagy proteins, including Atg18, Atg20, Atg21, and Atg24, to the phagophore assembly site, contributing to the growth of the phagophore [43]. During the expansion process, the phagophore is responsible for capturing cytoplasmic materials, subsequent maturation, and eventual closure. Two distinct protein conjugation processes are necessary for autophagosome formation during maturation. The first involves the conjugation of ATG5 to ATG12 catalyzed by ATG7 and ATG10. The second involves ATG7 and ATG3 facilitating the conjugation of the lipid phosphatidylethanolamine (PE) to microtubule-associated protein 1 LC3 (light chain 3) (ATG8) [44]. The multimeric complex of Atg12-Atg5-Atg16L serves as a mediator for the lipidation of LC3. Following autophagy initiation, cytosolic LC3 undergoes cleavage to form LC3-I, which subsequently converts to LC3-II, a form associated with autophagosomes. P62/SQSTM1 (sequestosome 1) is transported to autophagosomes, thereby initiating their biogenesis. As a result, autophagic flux occurs, encompassing the maturation of autophagosomes and subsequent degradation of trapped substrates by lysosomal enzymes. The fusion of autophagosomes and lysosomes is mediated by the interaction of soluble NSF-attachment protein (SNAP) receptors with their respective target proteins, including SNAP29 (synaptosome-associated protein), VAMP8 (vesicle-associated membrane protein-8), and STX17 (syntaxin-17) [45]. The lysosomal acid hydrolases degrade the cargo, with breakdown components being released into the extracellular matrix or returned to the cytosol for anabolic purposes [46]. A schematic representation of the core mechanisms of autophagy is given in Figure 1.

## 3. Autophagy Regulates Vascular Stem Cells

The homogeneity of composition and structure of the vascular niches is maintained by stem cells (SCs), ensuring a gradual renewal of cell elements and their subsequent repair in case of damage. Pericardial cells are present in small numbers and are characterized by three main functions [2,7,47]. These include the abilities to self-renew, to undergo uni- or multipotent differentiation in the direction of vascular predisposition cells, and to enter a “quiescent” state and be activated by an appropriate stimulus. These processes activate asymmetric division and ensure the reproduction of SCs and the formation of their precursors. Professor Beltrami and colleagues were the first to confirm the presence of vascular SCs in the heart and describe a population of c-kit+ cardiac stem cells [48]. Vasculogenic stem cells in the adult human heart exhibit c-kit and KDR receptor expression on the cell surface. These cells are localized in the perivascular space and can self-renew and undergo clonogenic and multipotent differentiation towards ECs and SMCs [49]. Recent studies using transgenic animal and ESC differentiation models have identified other populations of SCs characterized by the expression on the surface, including Sca1, Abcg2 (side population), as well as embryonic heart markers (e.g., Isl1 or Bmi1) [7,50,51,52]. Although a comprehensive description of the microenvironment for all types of SCs is still lacking, researchers generally agree on certain characteristics of their niche. These include low levels of oxidative damage and oxygen, as well as a preference for localization in the atria and cardiac apex, where hemodynamic stress is minimal [52,53,54]. Regional hypoxia has been shown to affect the behavior of cardiac SCs [55,56]. When cultured in an environment with low oxygen levels (<3%), cardiac progenitors were found to upregulate progenitor markers such as c-kit and BMI1 [57], positively impacting the survival, cardiac healing, cell migration, genomic stability, and vasculogenic potential of cardiac progenitor cells [58]. Additionally, there are reports indicating that the hypoxia-inducible factor may be involved in the induction of autophagy in SCs as a protective adaptative response to hypoxia. It is likely that there are alternative methods of energy generation and intracellular component recycling to ensure the self-renewal and viability of SCs in the surrounding environment, which is achieved through a basal level of autophagy. At the same time, it is worth noting that the excessive activation of autophagy can have a detrimental effect on SCs, as observed in cases of acute ischemic damage and oxidative stress induction. Reactive oxygen species (ROS) play a pivotal role in maintaining adult tissue homeostasis, profoundly influencing both vascular SCs and their progenitors. An increase in ROS levels has been demonstrated to induce autophagy dysfunction in SCs significantly. This is achieved by increasing the expression of autophagy formation markers LC3II/I and Beclin-1 and by triggering apoptosis through the ROS/NF-κB/NR4A2 signaling pathway. The inhibition of autophagy by bradykinin [59], miR-143 via Atg7 targeting [60], and autophagy inhibitors have been demonstrated to reverse the damage to SCs [61]. Thus, autophagy has been recognized as a critical intracellular regulator of cellular activities, including the survival and apoptosis of SCs.

## 4. Autophagy Is a Switcher of Vascular Cells Phenotype

Numerous studies have provided substantial evidence indicating that the development of vasculogenic dysfunction is associated with the entry of a subset of ECs into endothelial-mesenchymal transition (EndMT), leading to the acquisition of mesenchymal characteristics. These cells demonstrate typical morphology and function of mesenchymal cells, including increased motility and contractile properties [62]. ECs that have undergone EndMT lose the expression of endothelial cell proteins, including the CD31/platelet-endothelial cell adhesion molecule and von Willebrand factor, as well as VE-cadherin. Concurrently, they initiate the expression of mesenchymal cell proteins, including muscle α-smooth muscle actin, EDA fibronectin, N-cadherin, vimentin, fibroblast-specific protein-1, and type I and III fibrillar collagens. EndMT can be triggered in response to various profibrotic and pro-inflammatory stimuli that disrupt the physiological regulation of ECs [62,63]. Autophagy has been demonstrated to inhibit cellular transformation and promote the return of the endothelial cell phenotype. Following autophagy inhibition, microvascular ECs acquired a spindle-shaped morphology, increased secretory activity, and lost endothelial markers (CD31 and VE-cadherin). This was associated with the activation of the IL-6/MAPK/SNAIL signaling pathway and indicated signs of mesenchymal transformation. It is noteworthy that the neutralization of IL6 in vivo has been observed to suppress EndMT and result in the restoration of endothelial characteristics [64]. A comparable outcome was observed in HUVECs (human umbilical vein endothelial cells), wherein autophagy suppression triggered EndMT in an IL6-dependent manner [65]. Conversely, the stimulation of autophagy in HUVECs that had undergone EndMT resulted in the return of an endothelial cell phenotype, thereby promoting a mesenchymal-endothelial transition [66].

Additionally, autophagy has been demonstrated to be involved in the process of switching the phenotype of SMCs. In a healthy vascular microenvironment, SMCs display a contractile phenotype characterized by the expression of contractile proteins, including α-smooth muscle actin, smooth muscle protein 22-alpha, smooth muscle myosin heavy chain, and myocardin. These cells are responsible for regulating the tension and thickness of blood vessels. A variety of pathological factors, including inflammation, cytokines, signaling pathways, elements of the extracellular matrix (ECM), microRNAs, and biomechanical stresses, have been demonstrated to promote the switching of the phenotype of SMCs from the baseline contractile state to a proliferative synthetic state [67]. The platelet-derived growth factor PDGF-BB has been demonstrated to facilitate the contraction-synthesis phenotype switching of rat aortic SMCs and the induction of autophagy machinery [68,69]. Furthermore, the acquisition of synthetic phenotypes was prevented by the pharmacological inhibition of autophagy [69]. The synthetic phenotype of SMCs plays a pivotal role in the pathogenesis of cardiovascular disease through the overproduction of extracellular matrix and inflammatory cytokines, ultimately leading to inflammation and endothelial dysfunction. Furthermore, the excessive accumulation of these transformed cells also causes the thickening and narrowing of the vascular wall, playing a crucial role in vascular pathology.

## 5. Autophagy Regulates Cell-Cell Interactions, Endothelial Barrier Stability and Inflammation State in Vascular Niches

Intercellular contacts are crucial for various developmental processes, such as morphogenesis and the maintenance of tissue homeostasis. Three distinct types of contact complexes can be formed along the lateral surfaces of adjacent ECs: tight junctions, adhesive junctions, and desmosomes. Tight junctions are unique complex structures comprising at least 40 distinct proteins. They are composed of transmembrane proteins that bind to the cell membranes and the associated cytoplasmic proteins, forming direct membrane contact. This occurs in ECs and is involved in intracellular interaction and signal transmission. The extracellular domains of this multiprotein complex are directly connected to each other to form a network of sealing filaments, each of which operates independently of the others. These filaments control paracellular permeability and regulate the apical-basal membrane diffusion of membrane proteins, thereby maintaining cell surface polarity.

A substantial body of research supports the role of autophagy in regulating tight junctions and ensuring the integrity of the endothelial barrier. The research group headed by Professor Lee examined the impact of autophagy on tight junctions by analyzing the expression of ZO-1, a pivotal component of the complex structure [70,71]. The results demonstrated that LPS treatment led to an increase in autophagy markers, a reduction in ZO-1, and an enhancement in cell barrier permeability. LPS significantly reduced the expression of ZO-1, and this effect was further exacerbated by the inhibition of ATG7 or ATG5 expression. Furthermore, the inhibition of autophagy by chloroquine did not affect ZO-1 expression in control cells (human pulmonary microvascular ECs), but a significant decrease was observed following exposure to LPS [71].

Similarly, Lee et al. demonstrated that pretreatments with rapamycin and lithium carbonate resulted in a significant reversal of the decreased level of tight junction protein ZO-1 induced by oxygen-glucose deprivation/reoxygenation, accompanied by an enhanced distribution of ZO-1 on cell membranes [72]. In contrast, 3-MA pretreatment yielded disparate outcomes in both in vitro and in vivo contexts. Furthermore, autophagy plays a role in the regulation of the pro-inflammatory phenotype of ECs and the organization of adhesive junctions. The exposure of ECs to inflammatory stimuli results in the activation of the NF-κB signaling pathway. This process leads to the acquisition of a pro-adhesive and pro-inflammatory phenotype by ECs, which in turn promotes the expression of adhesion molecules (intercellular adhesion molecule-1 (ICAM-1) and vascular cell adhesion molecule-1 (VCAM-1) and E-selectin), cytokines (tumor necrosis factor-alpha (TNF-α) and interleukin-1 beta (IL-1β), interleukin-6 (IL-6)), and chemokines (interleukin-8 (IL-8) and monocyte chemoattractant protein-1 (MCP-1)) [73,74,75,76,77,78]. Professor Leonard demonstrated that Beclin-1 plays a role in regulating the inflammatory phenotype of human pulmonary artery ECs and in preventing endothelial barrier dysfunction by preventing the reassembly of adhesive junctions [79]. Following the silencing of Beclin-1, a notable reduction in NF-κB activity was observed in ECs, accompanied by impaired nuclear translocation and phosphorylation of RelA/p65. Furthermore, Beclin-1-downregulated ECs demonstrated enhanced barrier function as a consequence of augmented VE-cadherin reassembly at AJs and diminished actin stress fiber formation in response to thrombin stimulation. In conclusion, the presented findings reveal a previously unknown role of autophagy in the remodeling of EC junctions and the expression of key EC adhesion molecules. This facilitates intracellular trafficking and degradation [79,80].

Leukocyte transendothelial migration frequently occurs via the movement of leukocytes across junctions between adjacent ECs, which is regulated by the reorganization of junctionally expressed adhesion molecules. These include platelet endothelial cell adhesion molecule-1 (PECAM-1), members of the junctional adhesion molecule (JAM) family, and VE-cadherin [81,82,83]. The silencing of ATG5 in HUVECs has been observed to promote the formation of hotspot regions, which exhibit exaggerated paracellular neutrophil transport and increased neutrophil transendothelial migration [80]. Therefore, the inhibition of autophagy was observed to be associated with an increase in neutrophil infiltration within the perivascular microenvironment. As a consequence of the excessive neutrophil extravasation observed in the ATG5-deficient barrier, there was an associated enhancement in tissue damage. Furthermore, the neutrophil trafficking was suppressed by the local pharmacological induction of autophagy in ECs. Consequently, these results suggest the activation of EC autophagy as a potential anti-inflammatory strategy. Recent studies have demonstrated that autophagy can regulate the transcription, processing, and secretion of a number of inflammatory-associated cytokines. In particular, the disruption of normal autophagic pathways has been linked to the increased secretion of the pro-inflammatory cytokines IL-1a and IL-1b [84,85,86,87,88]. Similar to IL-1 cytokines, IL18 undergoes a secretory route, as the inhibition of autophagosome fusion or formation by bafilomycin A1 or ATG5 knockdown attenuates IL18 secretion [89]. In addition to the IL-1 family, other interleukins, particularly IL6 and CXCL8, have been demonstrated to be significantly associated with autophagy, which may contribute to the balance of pro-/anti-inflammatory cytokines in vascular microenvironment [90]. It has, therefore, been demonstrated that autophagy is implicated in regulating the permeability of the endothelial barrier, cell migration, and the release of pro-inflammatory factors.

## 6. Autophagy Regulates Cell-ECM Interactions in Perivascular Niche Cells

ECM is a complex three-dimensional network. It consists of collagen, elastin, glycoproteins, and proteoglycans. ECM plays a central role in regulating cell attachment and positioning, their interactions with regulatory molecules, such as growth factors and others, and the precise control of cell functions within the microenvironment. In both normal and pathological conditions, mechanical and chemical signals from the extracellular matrix are sensed by integrin-mediated adhesions, also known as focal adhesions [91,92]. This supramolecular complex serves to physically connect ECM to the actin cytoskeleton through an intricate plaque of proteins, also known as the adhesome network, organized in three distinct layers. The first is a signaling layer composed of transmembrane integrins and adaptor proteins, such as paxillin. The intermediate force-transduction layer comprises mechanotransduction molecules (e.g., talin and vinculin) and signaling molecules (e.g., FAK, Src, and PI3K). The final actin-regulatory layer is composed of actin and actin linker proteins (e.g., filamin and α-actinin) [91,93]. Cell–matrix interactions continuously and dynamically transform, significantly influencing a multitude of biological functions, including adhesion, cohesion, proliferation, differentiation, migration, and others. These interactions also have a profound impact on cellular phenotypes, significantly affecting the organization of the vascular niche.

The specific responses mentioned above can be achieved through the assembly and disassembly of focal contacts, determining the mobility and interaction with the matrix of cells within the vascular niche. One of the targets of autophagy is integrin β1, which is responsible for regulating the dynamics of adhesion contacts and promoting their turnover [94,95]. These processes occur via various pathways involving LC3 and autophagy receptors that target specific focal adhesion (FA) components. For example, the selective autophagy cargo adaptor NBR1 can bind to various FA proteins, including vinculin, FAK, paxillin, and zyxin, and recruit LC3-containing autophagosomes to FAs. Furthermore, numerous known mediators of integrin signaling, including the Src family kinase Fyn, FAK, p130Cas, and paxillin, have been demonstrated to regulate autophagy [95,96,97,98].

An alternative regulatory pathway may be the interaction with soluble matrikines that differentially modulate the autophagic response as part of the cellular microenvironment. It is noteworthy that decorin, endostatin, endorepellin, collagen VI, and kringle 5 are pro-autophagic matrix components that engage a variety of cell surface receptors, such as VEGFR2 [99,100], a5b1 integrin [100], a2b1 integrin [101], and GRP78 (glucose-regulated protein) [102], while others exhibit opposing actions that are crucial for maintaining a specific level of autophagic activity.

It can be postulated that the cellular composition of the matrix may undergo dynamic alteration within the perivascular microenvironment, with the direct involvement of autophagy. This can be achieved through secretory autophagy, which has been demonstrated to regulate the production of collagen I, fibronectin, and periostin [103]. Another hypothesis is that autophagy may regulate cell–matrix interactions by secreting proteolytic enzymes that induce matrix remodeling. A number of studies have identified autophagy as a key regulator of matrix metalloproteinases (MMPs) and cathepsins (CTS) secretion in different cell types [104,105,106,107]. The inhibition of autophagy by the depletion of ATG7 or ATG12 has been demonstrated to reduce the secretion of MMP-2 in conditioned media, thereby impairing their invasion ability [108]. Furthermore, it has been reported that autophagic flux regulates the secretion of CTS in macrophages, evidenced by the observation that the knockdown of ATG genes and 3-MA treatment resulted in reduced CTS secretion [109,110]. Concurrently, autophagy enhances the expression of MMP-2 and MMP-9, which is associated with the condition of the glycocalyx on the HUVECs surface [111]. The secretion of these proteases by autophagy is crucial for remodeling the perivascular microenvironment.

## 7. Autophagy Regulates Endothelial Cell Function and Angiogenesis

The term “vascular niche” has been defined as the microenvironment created by vascular ECs, which influences the behavior of cells in the vascular wall, the perivascular space, and the internal circulatory system. The crucial function of ECs in this system is to ensure the establishment of fundamental principles of interaction and cellular niche functionality. The number of studies confirming the involvement of autophagy as the primary regulator of the endothelium is on the rise, with new research emerging annually. Autophagy does play a detrimental role in maintaining the equilibrium and dynamic change in the status of ECs from quiescence to proliferation and sprouting. Recent studies have demonstrated that the activation of angiogenic behavior in ECs occurs through the elevation of autophagic flux via the AMPK-dependent mechanism [112]. Vascular endothelial growth factor (VEGF) has been identified as a potent agonist of AMPK activation, with the catalytic isoform AMPKα1 being shown to play a pivotal role in VEGF-induced angiogenesis [113,114,115]. One potential explanation is that AMPK inhibits glutamine:fructose-6-phosphate amidotransferase 1 (GFAT1), resulting in the reduced formation of O-linked β-N-acetylglucosamine-modified proteins. These proteins typically negatively influence angiogenesis. Another potential is for AMPK to activate endothelial nitric oxide synthase (eNOS) and promote angiogenesis through enhanced NO production [113,116,117,118,119,120]. In addition, studies have revealed an important role of autophagy in regulating nitric oxide (NO) bioavailability [121,122,123,124]. NO is a key molecule in the vasculature, regulating blood flow and vascular tone. eNOS phosphorylation is impaired, and NO generation in response to shear stress is decreased when autophagy is inhibited in ECs through ATG3 knockdown [121]. Inducing autophagy in ECs subjected to constant laminar shear stress elevates eNOS expression and enhances vascular tone [125]. These data confirm the participation of autophagy in the regulation of vasomotor responses.

Furthermore, ATG5 deficiency resulted in alterations in both VEGF/VEGFR2 and HGF/MET signaling in HUVECs, indicating that autophagy may be a crucial factor in the proper functioning of multiple tyrosine kinase receptors. A comparable impairment in VEGFR2-eNOS signaling was observed in primary lung ECs [126]. Conversely, the depletion of the autocrine VEGF signal from the endothelium results in FOXO1-mediated mitochondrial fragmentation and the suppression of glucose metabolism, leading to increased autophagy and ultimately contributing to cell death [127]. Marked autophagy was induced in microvascular ECs in response to hypoxia/reoxygenation, and the attenuation of this process resulted in a decrease in hypoxia/reoxygenation-induced injury through the upregulation of mammalian target of rapamycin (mTOR) phosphorylation. It was demonstrated that ischemia/reperfusion (I/R) induced p65-Beclin-1-dependent autophagic death in HUVECs, indicating that I/R autophagy exacerbates myocardial injury [128]. Similarly, endothelial cell dysfunction in obese rats with hypertension is attributable to excessive autophagy. It is noteworthy that autophagy is involved in the protective mechanisms of bioactive drug candidates against cardiovascular diseases. Epigallocatechin gallate, a major polyphenol in green tea, has been demonstrated to enhance autophagy in bovine aortic ECs, thereby reducing lipid accumulation. The activation of autophagy was found to mediate the atheroprotective effects of gossypetin, a natural compound, against ox-LDL-induced injury in HUVECs [129]. Moreover, mitophagy involving PTEN-induced kinase 1 and PARKIN is activated in response to metabolic stress in ECs and prevents mitochondrial dysfunction and metabolic stress-induced endothelial injury [130,131]. Furthermore, autophagy not only exerts cytoprotective effects in vascular niches but also contributes to the generation of senescent ECs that release SASP factors and inflammatory cytokines, leading to detrimental effects on neighboring cells in the microenvironment [132]. This process contributes to the development and progression of vascular diseases. Studies conducted on endothelium-specific ATG5-knockout mice and obese mice have provided evidence to support the dependence of the SASP ECs phenotype on the mTOR/autophagy pathway [132,133,134]. The activation of autophagy has been demonstrated to reduce inflammation, thereby inhibiting the SASP in senescent ECs through the regulation of autophagy. Therefore, it can be concluded that endothelial autophagy has a significant cytoprotective function and regulates the essential angiogenic characteristics of ECs by modulating its activity in response to stress.

## 8. Conclusions

**Figure 2:**
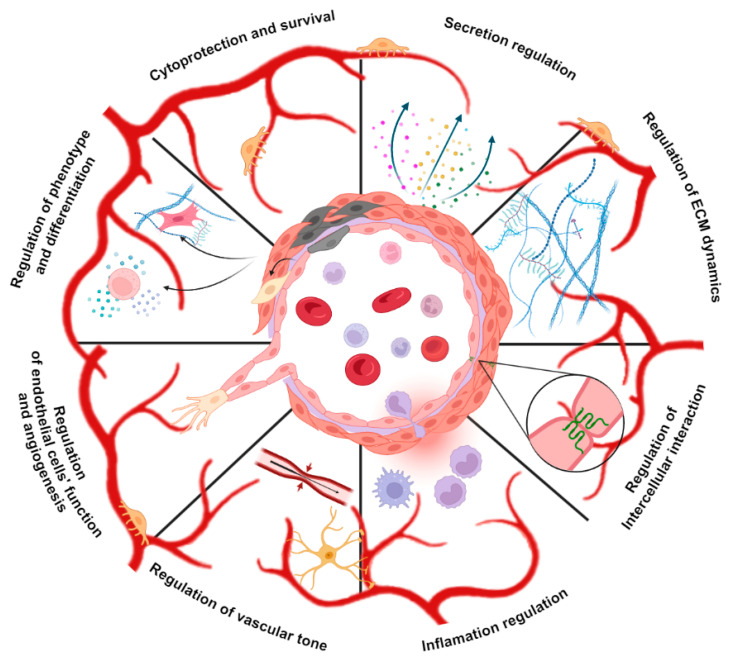
Autophagy regulates pivotal processes associated with vasculature niche homeostasis. Created with BioRender.com (https://app.biorender.com accessed on 21 August 2024).

The study of the principles of organization and composition of the vascular microenvironment has been at the peak of interest for the last few decades. However, the structural, functional, and molecular mechanisms remain poorly understood. This is due to the inherent challenges of studying the microenvironment and simulating it ex vivo, as well as the intricacy of signaling interactions that vary significantly across different tissues and in response to pathological insults. Moreover, no specific therapeutic intervention targeting vascular niches has been demonstrated to be efficacious in large-scale randomized clinical trials for the treatment of vasculopathy. Here, we provide an up-to-date review of experimental evidence that suggests a significant modulatory role of autophagy in the regulation of vascular niche functions (Figure 2). These findings could be of great significance in both basic and clinical research and facilitate the development of innovative therapeutic strategies for vascular diseases. The understanding of regulatory networks controlling autophagic induction, maintenance, and termination is quickly advancing, revealing their significance in numerous cellular processes, including metabolism, growth, proliferation, differentiation, genome stability, and apoptosis. A number of pharmacological and nutritional interventions have been identified as potential therapeutic candidates for patients with vascular diseases, such as mTORC1 inhibitors, AMPK activators, caloric restriction, natural compounds, and specific miRNAs. Enhancing our understanding of the physiological mechanisms that regulate autophagy through further studies will facilitate the development of new approaches to stimulate regenerative processes, maintain the homeostasis of vascular niches, and correct vascular dysfunction.

## Data Availability

Not applicable.
